# Membrane-Bound Transcription Factor ZmNAC074 Positively Regulates Abiotic Stress Tolerance in Transgenic *Arabidopsis*

**DOI:** 10.3390/ijms242216157

**Published:** 2023-11-10

**Authors:** Yexiong Qian, Yan Xi, Lingxue Xia, Ziling Qiu, Li Liu, Hui Ma

**Affiliations:** 1 Anhui Provincial Key Laboratory of Conservation and Exploitation of Important Biological Resources, College of Life Sciences, Anhui Normal University, Wuhu 241000, China; 2 Key Laboratory of Rice Genetic Breeding of Anhui Province, Rice Research Institute, Anhui Academy of Agricultural Sciences, Hefei 230031, China

**Keywords:** maize, *ZmNAC074*, abiotic stress, transcription factor

## Abstract

Maize (*Zea mays* L.) is one of the most widely cultivated crops for humans, making a vital contribution to human nutrition and health. However, in recent years, due to the influence of external adverse environments, the yield and quality of maize have been seriously affected. NAC (NAM, ATAF1/2 and CUC2) transcription factors (TFs) are important plant-unique TFs, which are crucial for regulating the abiotic stress response of plants. Therefore, it is of great biological significance to explore the underlying regulatory function of plant NAC TFs under various abiotic stresses. In this study, wild-type and *ZmNAC074*-overexpressed transgenic *Arabidopsis* were used as experimental materials to dissect the stress-resistant function of *ZmNAC074* in transgenic *Arabidopsis* at phenotypic, physiological and molecular levels. The analyses of seed germination rate, survival rate, phenotype, the content of chlorophyll, carotenoids, malondialdehyde (MDA), proline and other physiological indexes induced by distinct abiotic stress conditions showed that overexpression of *ZmNAC074* could confer the enhanced resistance of salt, drought, and endoplasmic reticulum (ER) stress in transgenic *Arabidopsis*, indicating that *ZmNAC074* plays an important regulatory role in plant response to abiotic stress, which provides an important theoretical foundation for further uncovering the molecular regulation mechanism of *ZmNAC074* under abiotic stresses.

## 1. Introduction

Global agriculture is seriously threatened by various ecological and environmental conditions, resulting in substantial declines in crop yields. These yield losses are expected to increase further with global climate change [1]. Plants in terrestrial environments must face a myriad of unpredictable and extreme environmental challenges, particularly various abiotic stresses such as high temperature, drought, and high salinity, which greatly limit the distribution, growth, and development of plants [2,3]. The process of resisting stress in plants is complex and involves molecular, metabolic, and physiological levels [2,4,5]. When confronted with adverse environments, plants have evolved a series of adaptive strategies that can perceive environmental stress stimuli and thus respond to naturally occurring stress signals through specific regulatory mechanisms to improve plant tolerance [3,6,7].

In general, plants have developed distinct regulatory mechanisms to cope with different abiotic stresses [7]. For example, to adapt to the water deficiency in the soil, plants change their physiological function and then alter the growth and structure of the root system, and also close the leaf stomata in the aboveground part [8,9]. Furthermore, previous research has demonstrated that drought tolerance in plants is greatly influenced by redox regulation and antioxidant systems [10,11]. Similarly, plants have evolved appropriate mechanisms to adapt to salt environments, including regulating ion homeostasis, activating osmotic stress pathways, mediating plant hormone signals, and pathways dependent on reactive oxygen species (ROS) homeostasis [12,13,14]. In addition, when plants are stimulated by the external environment, it can lead to ER stress. Generally, under low ER stress, plants can positively regulate protein folding and degradation through the unfolded protein response (UPR), so as to restore the function of the ER [15,16].

Transcription factors are essential for plants to respond to a variety of environmental stresses [17,18,19]. Increasing studies have shown that specific NAC proteins not only participate in the regulation of plant growth and development, but also act as key regulatory factors to regulate various stress signaling pathways [20,21,22,23]. A substantial number of NAC family members are capable of responding to various abiotic stresses in plants [21,24]. For instance, overexpression of SNAC3 and ONAC066 can enhance the drought resistance of transgenic rice compared to wild-type rice [25,26]. The drought tolerance of wheat plants is weakened by knocking out *TaNAC071-A*, while overexpression of *TaNAC071-A* enhances drought resistance in wheat [27]. In addition, rice *ONAC022* can play a significant role in salt stress by regulating ABA in various ways [28]. Potato NAC053 overexpression in *Arabidopsis* may enhance salt tolerance of transgenic *Arabidopsis* by promoting the expression of stress-associated genes [29]. Moreover, the membrane-binding transcription factor NAC062 of *Arabidopsis* transmits ER stress signals from the plasma membrane to the nucleus and thereby activates and up-regulates the expression of UPR-associated genes [30]. The *NAC103* gene in *Arabidopsis* transmits transcriptional regulatory signals through the ER stress cis-element UPRE-III to downstream genes of UPR through its encoded protein, positively regulating ER stress [31].

Maize is one of the most widely cultivated crops and the most abundant grain crop in the world, and it also has become an important model monocot species for functional genomics analysis [32]. Studies have shown that drought or salt stress can seriously affect the growth and development of maize, which in turn affects the livelihood and economy of millions of people around the world [33]. Plant tolerance to various environmental stresses can be enhanced by NAC TFs, and thus they have potential use in crops to acquire stress tolerance and further develop stress-resilient varieties [34,35]. In our previous studies, *ZmNAC074* has been demonstrated to respond in maize to heat, drought and salt stresses, and it can enhance the heat tolerance of *ZmNAC074*-overexpressing transgenic *Arabidopsis* [36]. In this study, *ZmNAC074*-overexpressing transgenic *Arabidopsis* were further used as experimental materials to dissect the underlying regulatory function of *ZmNAC074* under salt, drought, and ER stress from the phenotype, physiology, and molecular levels. This study will provide an important basis for an in-depth exploration of the stress resistance function and molecular regulation mechanism of *ZmNAC074* in maize.

## 2. Results

### 2.1. Germination of ZmNAC074-Overexpressing Transgenic Arabidopsis Seeds under Salt Stress

**Figure 1:**
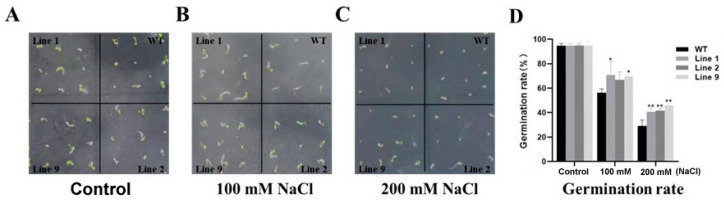
Germination of WT and transgenic *Arabidopsis* under salt stress. (**A**–**D**) The phenotype and germination percentage of *Arabidopsis* under 0 (control), 100 and 200 mM NaCl treatments. The error line (T) represents the standard deviation (±SD). Asterisks indicate significant differences, that is, * for *p* < 0.05 and ** for *p* < 0.01.

To investigate the germination rate of wild-type and transgenic *Arabidopsis* under salt stress, wild-type and transgenic *Arabidopsis* were seeded on 1/2 MS plates with 0, 100, and 200 mM NaCl, and the germination rate was counted seven days after vernalization. Under normal conditions, there was no significant difference in the germination rates of all *Arabidopsis* seeds, and the germination rates were all close to 95% (Figure 1A,D). Under 100 mM NaCl treatment, the germination rate of transgenic Line 1 and 9 was significantly higher than that of the wild-type (Figure 1B,D). In addition, when the NaCl concentration reached 200 mM, although the germination rates of all wild-type and transgenic *Arabidopsis* lines were lower than 50%, the germination rate of transgenic *Arabidopsis* lines was still higher than that of the wild-type (Figure 1C,D). In addition, under the two concentrations of salt stress, except for transgenic Line 9, which had a small number of green leaves after germination, the other lines hardly grew any green leaves. In conclusion, under salt stress, *ZmNAC074* in *Arabidopsis* can improve the germination rate of transgenic *Arabidopsis* seeds.

### 2.2. The Phenotype of ZmNAC074-Overexpressing Transgenic Arabidopsis Seedlings under Salt Stress and the Determination of Physiological Indexes

**Figure 2:**
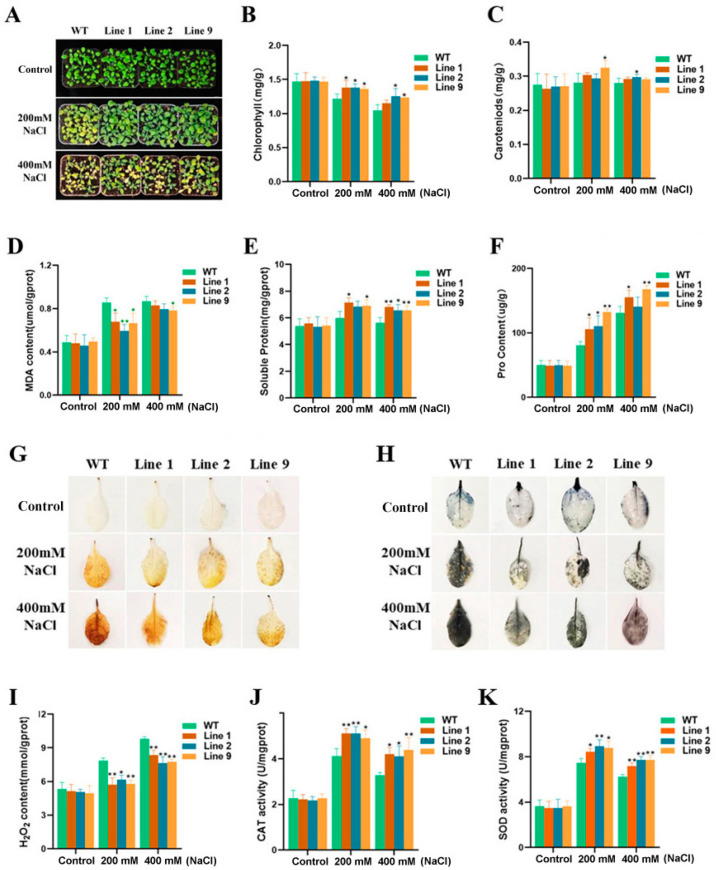
Analysis of salt tolerance of transgenic *Arabidopsis*. (**A**) Phenotypes analysis of WT and transgenic *Arabidopsis* under 0 (control), 200 and 400 mM NaCl treatments. (**B**–**F**) The content of chlorophyll, carotenoid, MDA, soluble protein and proline of *Arabidopsis* under salt stress. (**G**,**H**) DAB and NBT staining. (**I**–**K**) H_2_O_2_ content and SOD, CAT activity before and after salt stress treatment. The error line (T) represents the standard deviation (±SD). Asterisks indicate significant differences, that is, * for *p* < 0.05 and ** for *p* < 0.01.

To further determine the relationship between *ZmNAC074* and salt tolerance of transgenic *Arabidopsis*, WT and transgenic *Arabidopsis* were treated with 200 and 400 mM NaCl for 10 days. Before salt stress treatment, there was no significant difference in the phenotype between transgenic *Arabidopsis* and WT (Figure 2A). However, under 200 mM NaCl stress treatment, the leaves of the WT showed obvious yellowing, but only a few of the leaves of transgenic *Arabidopsis* yellowed (Figure 2A). Under 400 mM NaCl stress treatment, the leaves of the WT and overexpression lines were yellowed in most plants, but the leaves of the WT also showed withering largely, while the yellowing degree of leaves of transgenic *Arabidopsis* was significantly lower than that of WT *Arabidopsis*. The above phenotypic observations showed that the salt tolerance of transgenic *Arabidopsis* was stronger than that of WT *Arabidopsis*.

To further explore the factors that may affect the salt tolerance of transgenic *Arabidopsis*, the physiological indexes of some *Arabidopsis* leaves under normal conditions and under 200 and 400 mM NaCl stress treatments were measured, respectively. Under normal conditions, there was no significant difference in the contents of chlorophyll, carotenoid, MDA, soluble protein, and proline between the wild-type (WT) and transgenic *Arabidopsis* (Figure 2B–F). However, after treatment with two different concentrations of salt stress for 10 days, except that there was no significant difference in chlorophyll content between Line 1 and WT under 200 mM NaCl stress treatment, the chlorophyll content of other transgenic lines was significantly higher than that of WT *Arabidopsis* (Figure 2B,C). In addition, except for the carotenoid content of Line 9 under 200 mM NaCl treatment and Line 2 under 400 mM NaCl treatment being higher than that of WT *Arabidopsis*, there was no significant difference in carotenoid content between WT and transgenic *Arabidopsis* under other salt stress treatments. Furthermore, the MDA content of transgenic *Arabidopsis* under 200 mM NaCl treatment was significantly lower than that of the WT (Figure 2D), while under 400 mM NaCl stress, only the MDA content of Line 9 was significantly lower than that of WT *Arabidopsis*. Additionally, except for the proline content of Line 2 under 200 mM NaCl treatment and the soluble protein content of Line 2 under 400 mM NaCl treatment not being significantly different from those of WT *Arabidopsis*, the proline and soluble protein contents of transgenic lines under other salt stress treatments were significantly higher than those of WT *Arabidopsis* (Figure 2E,F). To conclude, overexpression of *ZmNAC074* enhanced the salt tolerance of transgenic *Arabidopsis*.

The leaves of all *Arabidopsis* lines under 200 and 400 mM NaCl stress were stained with DAB and NBT, respectively (Figure 2G,H). Under normal conditions, there was no significant difference in leaf color stained with DAB and NBT between the WT and the transgenic *Arabidopsis* lines. However, the leaves of dyed WT and transgenic *Arabidopsis* lines became darker after 10 days of salt stress, indicating that the contents of H_2_O_2_ and O_2_^−^ increased under salt stress. In addition, the color of *Arabidopsis* leaves treated under 400 mM NaCl stress was darker than that under 200 mM NaCl stress. However, under either the treatment of 200 mM NaCl or 400 mM NaCl, the leaf color of the transgenic *Arabidopsis* lines was lighter than that of WT *Arabidopsis*, indicating that the contents of H_2_O_2_ and O_2_^−^ in the transgenic lines were lower than those in WT *Arabidopsis* under salt stress. Correspondingly, by measuring the content of H_2_O_2_, it was found that the content of H_2_O_2_ of transgenic *Arabidopsis* was indeed lower than that of WT *Arabidopsis* after salt stress (Figure 2I). Similarly, after the determination of CAT and SOD enzyme activity, the corresponding results were obtained, that is, under two concentrations of salt stress, the CAT and SOD enzyme activity of transgenic *Arabidopsis* was higher than that of WT *Arabidopsis* (Figure 2J,K). However, the activities of CAT and SOD in WT and transgenic *Arabidopsis* leaves treated with 400 mM NaCl were lower than those treated with 200 mM NaCl. Collectively, compared with WT *Arabidopsis*, the ROS level of transgenic *Arabidopsis* was lower, which further indicated that transgenic *Arabidopsis* had stronger salt tolerance.

### 2.3. Overexpression of ZmNAC074 Regulates the Expression of Stress-Responsive Genes under Salt Stress

**Figure 3:**
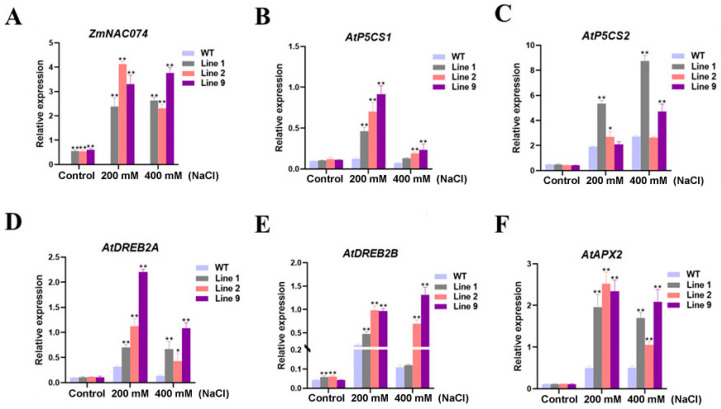
The expression of stress-responsive genes under salt stress in WT and transgenic *Arabidopsis*. (**A**–**F**) The expression of *ZmNAC074*, *AtP5CS1*, *AtP5CS2*, *AtDREB2A*, *AtDREB2B* and *AtAPX2*. The error line (T) represents the standard deviation (±SD). Asterisks indicate significant differences, that is, * for *p* < 0.05 and ** for *p* < 0.01.

To explore the possible role of *ZmNAC074* in salt stress, the expression of *ZmNAC074*, stress response genes and *AtAPX2* was determined, respectively (Figure 3). After salt stress, the expression of *ZmNAC074* in transgenic *Arabidopsis* was higher than that in normal conditions (Figure 3A). In addition, under 200 mM NaCl stress, the expression of *ZmNAC074* of Line 1 and 9 was slightly lower than that of Line 2, and the expression of *ZmNAC074* in Line 9 was the highest under 400 mM NaCl stress. However, compared with 200 mM NaCl treatment, the expression of *ZmNAC074* in transgenic *Arabidopsis* was down-regulated under 400 mM NaCl stress. In addition, under normal conditions, except that the expression of *AtDREB2B* in Line 1 and 2 was higher than that of WT *Arabidopsis*, there was no significant difference in the expression of other stress-responsive genes between the two *Arabidopsis* (Figure 3B–F). After salt stress, the expression of stress response genes and *AtAPX2* increased in all *Arabidopsis* lines, but the expression of stress response genes and *AtAPX2* under 200 mM NaCl stress was higher than that under 400 mM NaCl stress. In addition, after salt stress, the expression of *AtP5CS2* in Line 9 under 200 mM NaCl stress, *AtP5CS1* and *AtDREB2B* in Line 1 under 400 mM NaCl stress and *AtP5CS2* in Line 2 was not significantly different from those in WT *Arabidopsis*, but the expression of related genes in other transgenic lines was significantly higher than those in WT *Arabidopsis*. These results suggest that *ZmNAC074* may directly or indirectly regulate the expression of stress-responsive genes in transgenic *Arabidopsis* to enhance its salt tolerance.

### 2.4. Germination of ZmNAC074-Overexpressing Transgenic Arabidopsis Seeds under Drought Stress

**Figure 4:**
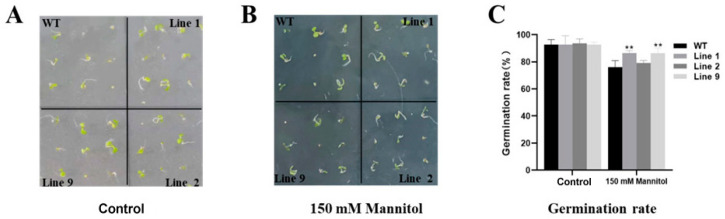
Germination of WT and transgenic *Arabidopsis* under normal (control) and mannitol treatment. (**A**,**B**) The germination phenotype of WT and transgenic *Arabidopsis* treated in drought simulation. (**C**) Statistics of germination percentage of *Arabidopsis* under drought stress treatment. The error line (T) represents the standard deviation (±SD). Asterisks indicate significant differences, that is, ** for *p* < 0.01.

To explore the effects of drought stress on the germination of WT and transgenic *Arabidopsis*, WT and transgenic *Arabidopsis* were seeded in 1/2MS plates containing 0 and 150 mM mannitol. Under normal conditions, the germination rates of WT and transgenic *Arabidopsis* lines were not significantly different after 7 days (Figure 4A,C). Under the treatment of 150 mM mannitol, the germination rate of transgenic Line 1 and Line 9 was significantly higher than that of WT *Arabidopsis* on the 7th day (Figure 4B,C). Based on the above results, the overexpression of *ZmNAC074* in *Arabidopsis* could improve the germination rate of *Arabidopsis* seeds under mannitol treatment.

### 2.5. Phenotypic of ZmNAC074-Overexpressing Transgenic Arabidopsis Seedlings under Drought Stress and Determination of Physiological Indexes

**Figure 5:**
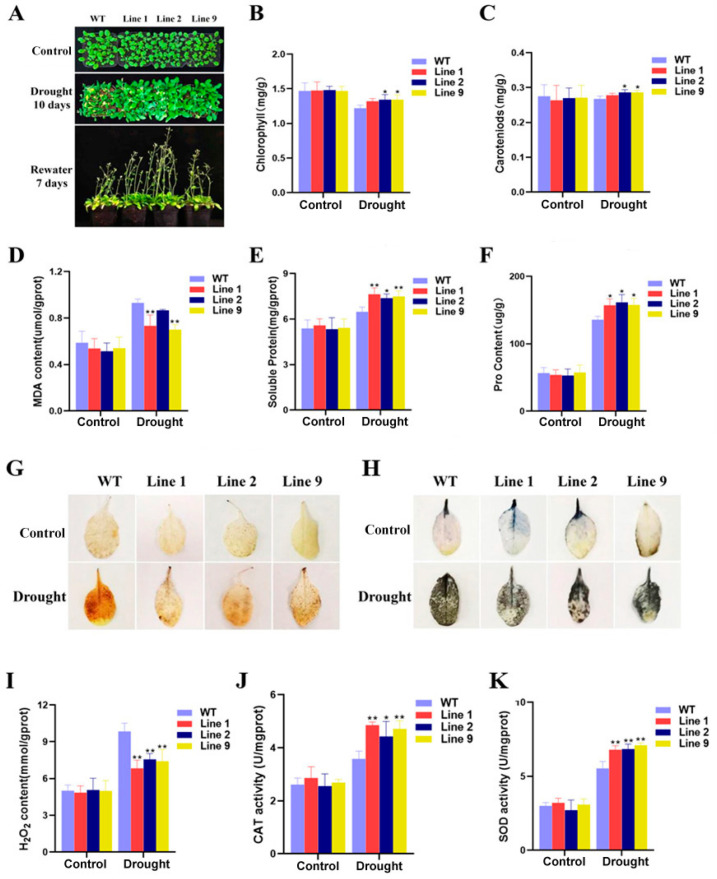
Drought tolerance analysis of transgenic *Arabidopsis*. (**A**) Phenotypic changes in WT and transgenic *Arabidopsis* under normal (control) and drought treatment. (**B**–**F**) The contents of chlorophyll, carotenoid, MDA, soluble protein and proline of *Arabidopsis* under drought stress. (**G**,**H**) DAB and NBT staining. (**I**–**K**) H_2_O_2_ content and SOD, CAT activity under water deficiency treatment. The error line (T) represents the standard deviation (±SD). Asterisks indicate significant differences, that is, * for *p* < 0.05 and ** for *p* < 0.01.

To further determine the relationship between *ZmNAC074* and drought tolerance of transgenic *Arabidopsis*, WT and transgenic *Arabidopsis* were subjected to a water deficit for 10 days, and then watered again for one week, respectively. It was observed that under water deficiency, the leaves of WT *Arabidopsis* showed large-scale curl and withering, and nearly half of the leaves showed obvious yellowing, while the overexpressed *Arabidopsis* lines only showed slight yellowing at the leaf tip, but the yellowing degree was not as deep as that of WT *Arabidopsis* (Figure 5A). After resuming watering, some leaves of WT *Arabidopsis* returned to normal, but most of the leaves of WT *Arabidopsis* were yellowed, while the leaves of transgenic lines were only slightly yellowed, and the degree of yellowing was much lower than that of WT *Arabidopsis*. In addition, the plant height of transgenic *Arabidopsis* was significantly higher than that of WT *Arabidopsis*, especially Line 2. According to the above phenotype and plant height analysis, the drought resistance of transgenic *Arabidopsis* was stronger than that of WT *Arabidopsis*. Under normal conditions, there was no significant difference in the contents of chlorophyll, carotenoid, MDA, soluble protein and proline between transgenic *Arabidopsis* and WT *Arabidopsis*. However, under water deficiency treatment, only the transgenic Line 1 had no significant difference compared with WT *Arabidopsis*, but the chlorophyll and carotenoid contents of Line 2 and 9 were higher than those of WT *Arabidopsis* (Figure 5B,C). In addition, under the condition of water deficiency, the MDA content of Line 2 was not significantly different from that of WT, but the MDA content of Line 2 and 9 was lower than that of WT *Arabidopsis* (Figure 5D). Moreover, the contents of proline and soluble protein in transgenic *Arabidopsis* were higher than those in WT *Arabidopsis* (Figure 5E,F). To sum up, the drought tolerance of transgenic *Arabidopsis* with the overexpression of *ZmNAC074* was stronger than that of WT *Arabidopsis*.

DAB and NBT chemical staining were carried out on *Arabidopsis* leaves under normal growth and water stress treatment (Figure 5G,H). Under normal conditions, there was no significant difference in leaf color among all *Arabidopsis* lines after DAB and NBT staining, but under water deficiency, the color of stained WT and transgenic *Arabidopsis* leaves became darker, indicating that the production of H_2_O_2_ and O_2_^−^ increased. However, the color of WT *Arabidopsis* leaves was still darker than that of transgenic *Arabidopsis*, indicating that the accumulation of H_2_O_2_ and O_2_^−^ of WT *Arabidopsis* was higher than that of transgenic *Arabidopsis* after drought stress. Correspondingly, by measuring the H_2_O_2_ content after drought stress, it was found that the content of H_2_O_2_ of the transgenic lines was lower than that of WT *Arabidopsis* (Figure 5I). Similarly, the activities of CAT and SOD in transgenic *Arabidopsis* were higher than those in WT *Arabidopsis* (Figure 5J,K). Collectively, the antioxidant capacity of transgenic *Arabidopsis* is stronger than that of WT *Arabidopsis*.

### 2.6. Overexpression of ZmNAC074 Regulates the Expression of Stress-Responsive Genes under Drought Stress

**Figure 6:**
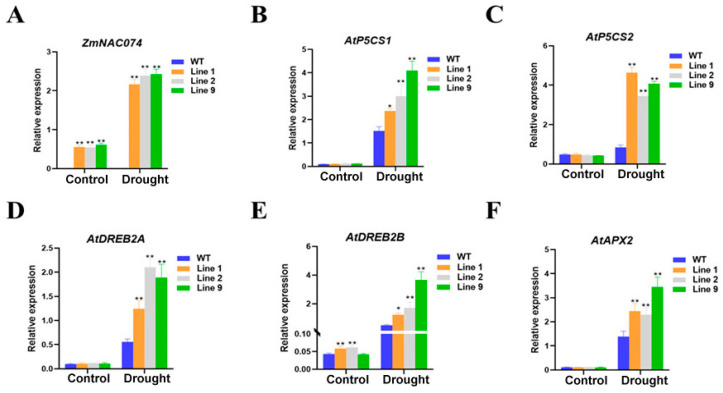
Analysis of the expression of stress-responsive genes under normal (control) and drought stress treatment. (**A**–**F**) The expression of *ZmNAC074*, *AtP5CS1*, *AtP5CS2*, AtDREB2A, *AtDREB2B* and *AtAPX2*. The error line (T) represents the standard deviation (±SD). Asterisks indicate significant differences, that is, * for *p* < 0.05 and ** for *p* < 0.01.

To explore the possible role of *ZmNAC074* under drought stress, the expression of some stress response genes and *AtAPX2* under normal conditions and after drought stress were determined (Figure 6). After drought stress, the expression of *ZmNAC074* in transgenic *Arabidopsis* increased significantly, but the level of Line 1 was slightly lower than that of Line 2 and 9 (Figure 6A). Under normal conditions, only the overexpression of *AtDREB2B* in *Arabidopsis* was higher than that of WT *Arabidopsis*, and there was no significant difference in the expression of other stress response genes compared with WT *Arabidopsis* (Figure 6B–F). Moreover, although the expression of these stress response genes and *AtAPX2* was up-regulated in all *Arabidopsis* after drought stress, the expression of these genes in transgenic *Arabidopsis* lines was significantly higher than that in WT *Arabidopsis* lines. To sum up, *ZmNAC074* may directly or indirectly activate and up-regulate the expression of stress-responsive genes in *Arabidopsis*, thus enhancing the drought tolerance of transgenic *Arabidopsis*.

### 2.7. Survival Rate of Transgenic Arabidopsis under ER Stress

**Figure 7:**
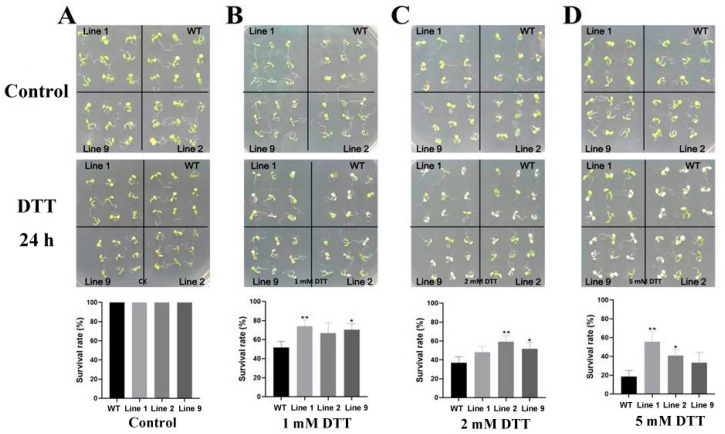
The phenotypic and survival rate of *Arabidopsis* under normal (control) and DTT treatment. (**A**–**D**) Phenotypic and survival rate of WT and transgenic *Arabidopsis* under 0, 1, 2 and 5 mM DTT treatments. The error line (T) represents the standard deviation (±SD). Asterisks indicate significant differences, that is, * for *p* < 0.05 and ** for *p* < 0.01.

WT and three transgenic *Arabidopsis* lines were grown in normal 1/2MS plates for 10 days, and then transplanted into 1/2MS plates containing 0, 1, 2 and 5 mM DTT for 24 h, respectively. The phenotypic changes were observed and the survival rate was counted (Figure 7). Before transplanting, there was no significant difference between WT and transgenic *Arabidopsis*. All *Arabidopsis* survived at 0 mM DTT (Figure 7A). After DTT treatment, a part of *Arabidopsis* leaves turned white, and the survival rate was calculated on the basis of keeping the green *Arabidopsis*. Under 1 mM DTT treatment, although there was no significant difference in the survival rate between Line 2 and WT, the survival rate of Line 1 and 9 was higher than that of WT *Arabidopsis* (Figure 7B). Under 2 mM DTT treatment, the survival rate of Line 2 and 9 was much higher than that of WT *Arabidopsis* (Figure 7C). In addition, although there was no significant difference in the survival rate between transgenic *Arabidopsis* Line 9 and WT *Arabidopsis* under 5 mM DTT treatment, the survival rate of transgenic *Arabidopsis* Line 9 was still higher than that of WT *Arabidopsis* (Figure 7D). Based on the above results, the overexpression of *ZmNAC074* in transgenic *Arabidopsis* can improve the survival rate of *Arabidopsis* seedlings under ER stress.

### 2.8. Phenotypic and Physiological Indexes of Transgenic Arabidopsis under ER Stress

**Figure 8:**
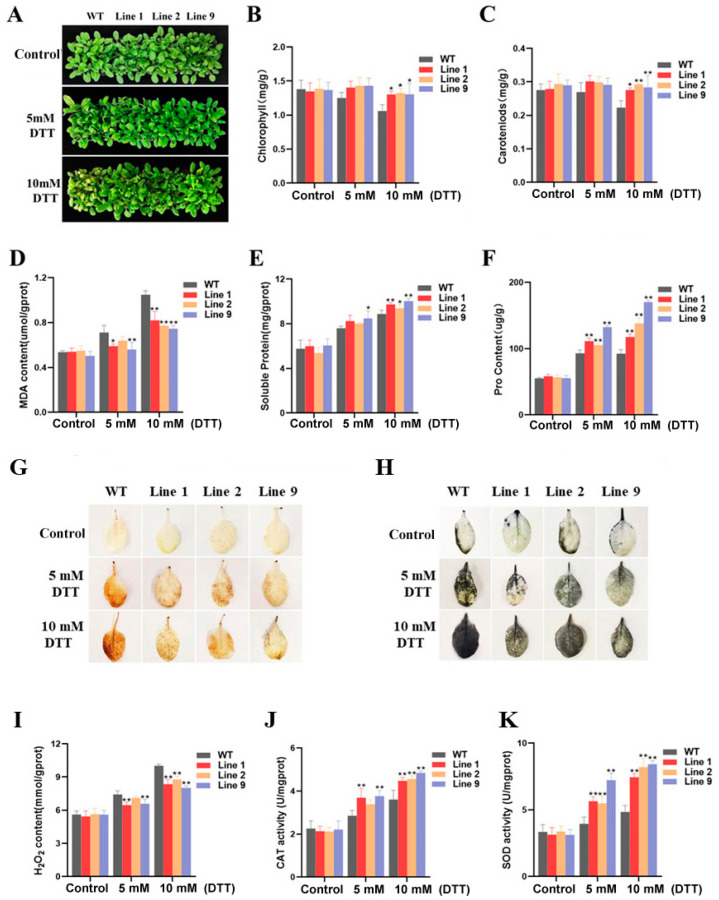
Analysis of the tolerance to RE stress in transgenic *Arabidopsis*. (**A**) Phenotype of WT and transgenic *Arabidopsis* under 0 (control), 5 and 10 mM DTT treatments, respectively. (**B**–**F**) The contents of chlorophyll, carotenoid, MDA, soluble protein, and proline of WT and transgenic *Arabidopsis* under DTT treatments. (**G**,**H**) DAB and NBT staining. (**I**–**K**) H_2_O_2_ content and the activity of SOD and CAT under DTT treatments. The error line (T) represents the standard deviation (±SD). Asterisks indicate significant differences, that is, * for *p* < 0.05 and ** for *p* < 0.01.

To further investigate whether *ZmNAC074* can improve the tolerance to ER stress in transgenic *Arabidopsis*, it was sprayed with 5 and 10 mM DTT for 24 h, respectively. It was observed that a large number of yellowish-brown spots appeared in the leaves of WT after DTT treatment, while there were only a few yellow brown spots in the leaves of transgenic lines, and the difference was more obvious in 10 mM DTT treatment (Figure 8A). Based on the above phenotypic observations, it is suggested that *Arabidopsis* with overexpression of *ZmNAC074* has stronger tolerance to ER stress than WT *Arabidopsis*.

To further explore the factors affecting the tolerance of *Arabidopsis* to ER stress, the contents of chlorophyll, carotenoid, MDA, soluble protein and proline under normal and two kinds of DTT treatment were measured, respectively (Figure 8B–F). Under normal conditions, there was no significant difference in the physiological indexes among all the WT and transgenic *Arabidopsis* lines. There was no significant difference in the contents of chlorophyll and carrots in the *Arabidopsis* lines under 5 mM DTT treatment for 24 h, but the chlorophyll and carotenoid contents of transgenic lines were significantly higher than those of the WT after 24 h of 10 mM DTT treatment (Figure 8B,C). In addition, under 5 mM DTT treatment, except that the MDA content of Line 2 was not significantly different from that of WT *Arabidopsis*, the content of MDA of other transgenic lines was lower than that of WT *Arabidopsis* (Figure 8D). Similarly, except that there was no significant difference in the soluble protein and proline content between transgenic *Arabidopsis* Line 1 and 2 under 5 mM DTT treatment and WT *Arabidopsis*, the soluble protein and proline contents of other transgenic lines under DTT treatment were higher than those of WT *Arabidopsis* (Figure 8E,F). In summary, the overexpression of *ZmNAC074* can better alleviate the ER stress in transgenic *Arabidopsis*.

All leaves of *Arabidopsis* under ER stress induced by 5 mM and 10 mM DTT were stained with DAB and NBT, respectively (Figure 8G,H). Under normal conditions, there was no significant difference in the color of the *Arabidopsis* leaves after staining. However, the color of stained WT and transgenic *Arabidopsis* leaves darkened significantly, indicating that the content of H_2_O_2_ and O_2_^−^ increased after 24 h of DTT treatment. The color of all *Arabidopsis* leaves under 10 mM DTT treatment was darker than that of 5 mM DTT treatment, but the color of WT *Arabidopsis* leaves was still darker than that of transgenic *Arabidopsis*. Correspondingly, by measuring the content of H_2_O_2_, it was found that except that the content of H_2_O_2_ of Line 2 under 5 mM DTT treatment was not significantly different from that of WT *Arabidopsis*, the content of H_2_O_2_ of other transgenic lines under DTT treatment was lower than that of WT (Figure 8I). After determining the activities of CAT and SOD, the corresponding results were also obtained, that is, after DTT treatment, the activities of CAT and SOD in transgenic *Arabidopsis* were significantly higher than those in WT *Arabidopsis* (except Line 2 under 5 mM DTT treatment) (Figure 8J,K). In summary, the overexpression of *ZmNAC074* may alleviate ER stress.

### 2.9. Expression Analysis of Stress-Responsive Genes in Transgenic Arabidopsis under ER Stress

**Figure 9:**
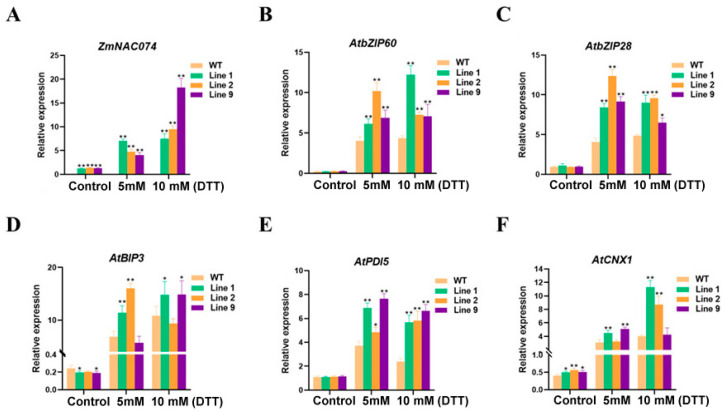
Changes in the expression of stress-responsive genes under normal (control) and DTT treatment. (**A**–**F**) The expression of *ZmNAC074*, *AtbZIP60*, *AtbZIP28*, *AtBIP3*, *AtPDI5* and *AtCNX1*. The error line (T) represents the standard deviation (±SD). Asterisks indicate significant differences, that is, * for *p* < 0.05 and ** for *p* < 0.01.

To clarify the possible role of *ZmNAC074* under ER stress, the expression of *ZmNAC074* and some stress response genes in WT and transgenic lines were determined (Figure 9). The expression of *ZmNAC074* in transgenic lines increased after DTT treatment. In addition, compared with 5 mM DTT treatment, the expression of *ZmNAC074* increased more than that of 10 mM DTT treatment, and the expression of *ZmNAC074* of Line 9 increased the most (Figure 9A). In addition, under normal growth conditions, except that the expression of *AtBIP3* and *AtCNX1* in transgenic lines was significantly higher than that in WT, the expression of other UPR-related genes in transgenic lines was not significantly different from those in WT (Figure 9B–F). After DTT treatments, the expression of all ER stress response genes increased, but the expression regularity of ER stress genes in two lines under DTT treatment was not strong. For example, the expression of *AtbZIP60* was the highest in Line 2 under 5 mM DTT treatment, but the overexpression Line 1 had the highest expression under 10 mM DTT treatment. The expression of *AtBIP3* in Line 9 under 5 mM DTT treatment and in Line 2 under 10 mM DTT treatment was slightly lower than that of WT treated with corresponding DTT concentration. However, the expression of UPR-related genes in transgenic *Arabidopsis* lines was higher than that in WT after DTT treatments. These results suggest that *ZmNAC074* may alleviate ER stress in transgenic *Arabidopsis*.

## 3. Discussion

Plants respond to abiotic stress in unique ways, including physiological and biochemical reactions, gene transcription, and a large number of stress-specific transcripts and metabolites [37]. Plants have evolved complex regulatory networks to adapt to adverse external environments [7]. Under abiotic stress, NAC TFs from various plants, such as ANAC087, NAC019 and AtNTL7 from *Arabidopsis* [38,39,40], OsNTL3 protein, SNAC3 and ONAC066 from rice [25,26,41] and TaNAC2L from wheat [42], can perform their functions under abiotic stress. In this study, the assays of seed germination rate at the plate stage and phenotypic identification of seedlings at the age of three weeks from the transgenic *Arabidopsis* overexpressing *ZmNAC074* were firstly performed to explore the underlying regulatory function of *ZmNAC074* under various abiotic stresses.

Further, some physiological indexes such as chlorophyll [43], MAD [44], proline [45] and ROS [4] are usually utilized to assess plant stress resistance. For example, MDA is the product of lipid peroxidation of biofilm, and the higher the content of MDA indicates the greater the degree of membrane damage in plant cells [44]. In this study, the transgenic *Arabidopsis* overexpressing *ZmNAC074* showed a lower MDA content compared to WT *Arabidopsis* under various abiotic stresses, indicating that *ZmNAC074* can enhance the stress resistance of transgenic *Arabidopsis* by reducing cellular lipid peroxidation. Moreover, proline can be utilized as a penetration agent or free radical scavenger to reduce cell damage [45]. For example, overexpression of *MfbHLH38* can increase the proline content of transgenic *Arabidopsis* under drought and salt stresses, which indicates that transgenic *Arabidopsis* exhibits stronger drought and salt tolerance than wild *Arabidopsis* [46]. In addition, *Arabidopsis* proline biosynthesis representative genes *AtP5CS1* and *AtP5CS2* can promote proline biosynthesis [47]. In this study, the transgenic *Arabidopsis* overexpressing *ZmNAC074* showed a large amount of proline accumulation under drought and salt stress compared to WT *Arabidopsis*, and the expression of *AtP5CS1* and *AtP5CS2* was also higher than that of WT *Arabidopsis*, indicating that *ZmNAC074* may enhance the stress resistance of transgenic *Arabidopsis* via promoting proline biosynthesis.

Furthermore, the degradation and synthesis of chlorophyll is impacted by abiotic stress [48]. Under abiotic stress, plants with a higher chlorophyll content can make better use of light energy for photosynthesis [43]. In addition, plant responses to environmental changes and stressors can be regulated by hormones and signaling molecules, including carotenoid-derived metabolites [2]. For example, sweet potato *IbARF5* has been revealed to be involved in carotenoid biosynthesis, salt and drought tolerance [49]. Moreover, carotenoids can also reduce the harmful heat-induced ROS [50]. In this study, the contents of chlorophyll and carotenoids in transgenic *Arabidopsis* were higher than those in WT *Arabidopsis* under abiotic stresses, indicating that *ZmNAC074* might enhance photosynthesis and stress resistance of transgenic *Arabidopsis* by promoting the synthesis of chlorophyll and carotenoids or reducing their degradation. However, the detailed mechanism for regulating photosynthetic pigments in transgenic *Arabidopsis* must be further explored by measuring the expression changes in certain chlorophyll and carotenoid synthesis and degradation genes.

Under stress conditions, the content of ROS increases rapidly, which disturbs the cell redox dynamic balance [51]. On the other hand, when the rate of ROS production decreases below the threshold level, it can regulate the redox signal pathway that promotes plant growth, development and adaptation to stress [52]. However, excessive ROS produced by various abiotic stresses is generally toxic and can cause oxidative damage to proteins, nucleic acids, cell membranes, lipids and so on [53]. In addition, SOD and CAT can protect plant cells from oxidative damage [51]. In this study, the transgenic *Arabidopsis* overexpressing *ZmNAC074* has a stronger ability to alleviate ROS damage under a variety of abiotic stresses. It can be speculated that *ZmNAC074* may be involved in modulating the level of ROS through ROS-mediated stress-responsive signal pathways to enhance the stress resistance of transgenic *Arabidopsis*. However, the specific relationship between *ZmNAC074* and ROS homeostasis needs to be further clarified, for example, by knocking out maize *ZmNAC074* to investigate the change in the ROS level in mutant plants.

In addition, the regulation of ROS levels usually involves antioxidant enzymes such as GPX, SOD and APX, which act as antioxidants and regulate oxidative signal transmission [54]. In this study, under abiotic stress, the expression of representative *AtAPX2* in transgenic *Arabidopsis* was significantly higher than that in WT *Arabidopsis*. This result is consistent with the cumulative content of ROS, indicating that *ZmNAC074* may improve the ROS scavenging ability of transgenic *Arabidopsis* by up-regulating the expression of some antioxidant enzyme genes, thus enhancing the stress resistance of transgenic *Arabidopsis*. In addition, other peroxidase activity and the expression of corresponding genes can be also determined to further clarify the mechanism of regulating ROS homeostasis in transgenic *Arabidopsis*.

The transcription factor AtDREB2A can activate the expression of downstream genes involved in drought and salt stress responses in *Arabidopsis* [55]. For example, drought-tolerant transgenic *Arabidopsis* with *MlNAC10* may be attributed to the up-regulated expression of *AtDREB2A* under drought and salt stresses [56]. In this study, under drought and salt treatments, the expression of *AtDREB2A* and *AtDREB2B* in transgenic lines was higher than that in WT *Arabidopsis*, indicating that *ZmNAC074* may trigger the expression of downstream genes in drought and salt stresses through the up-regulated expression of *AtDREB2A* and *AtDREB2B*, thus enhancing drought and salt tolerance in *Arabidopsis*.

Additionally, *AtbZIP60* encodes a UPR-associated transcription factor and can respond to dithiothreitol (DTT) and tunicamycin (TM), thus positively regulating the ER stress response [57]. Previous studies have revealed the close relationship between plant NAC proteins and ER stress [58]. For example, AtNAC062, AtNAC089 and AtNAC103 can be activated in response to ER stress and modulate different UPR-associated genes in *Arabidopsis* [30,31,59]. Similarly, it has been demonstrated that OsNTL3 protein in rice, which has a high homology with ZmNAC074 protein in maize, can directly bind to the promoter of the *OsbZIP74* gene and thereby regulate its expression in response to heat stress [41]. In this study, we found that the expression of *AtbZIP60* in transgenic lines treated with two concentrations of DTT was significantly higher than that of WT *Arabidopsis*, indicating that the ZmNAC074 protein may interact with the AtbZIP60 protein to alleviate ER stress. However, further experiments including yeast two hybrids are needed to reveal the interaction between them. In addition, UPR-associated genes *AtbZIP28*, *AtBIP3*, *AtPDI5* and *AtCNX1* in transgenic *Arabidopsis* were up-regulated in this study, and these genes can further modulate the expression of genes downstream of the UPR pathway [57,60,61,62]. Therefore, *ZmNAC074* may alleviate ER stress by regulating the expression of UPR-associated genes in transgenic lines, but the specific regulatory mechanism needs to be further elucidated, such as to determine the potential interaction between the *ZmNAC074* protein and UPR-associated proteins.

**Figure 10:**
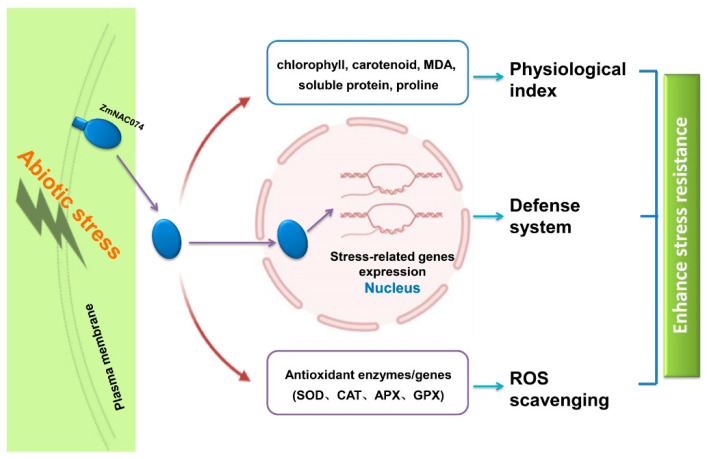
A proposed model for the potential roles of ZmNAC074 in response to abiotic stress in transgenic *Arabidopsis*.

Taken together, our results showed that ZmNAC074 is a stress-responsive NAC transcription factor in maize, as shown in the proposed model in this study (Figure 10). Phenotypic and physiological analyses displayed that overexpression of *ZmNAC074* in transgenic *Arabidopsis* confers enhanced abiotic stress tolerance significantly through modulating the accumulation of a variety of stress metabolites, including reactive oxygen species (ROS), antioxidants, malondialdehyde (MDA), proline, soluble protein, chlorophyll and carotenoid. Further, quantitative real-time PCR analysis revealed that the expression levels of some stress-responsive genes in transgenic *Arabidopsis* were significantly up-regulated under various abiotic stress treatments, suggesting that *ZmNAC074* may function as a positive regulator that triggers the expression of stress-associated genes to enhance plant stress tolerance under various abiotic stress conditions.

## 4. Materials and Methods

### 4.1. Plant Material and Growth Conditions

The *ZmNAC074*-overexpressing transgenic *Arabidopsis* lines used in this study were generated from our previously obtained seeds kept in our laboratory via transgenic methods. The *Arabidopsis* ecotype Columbia (Col) was used as original material for genetic transformation to obtain these transgenic plants and perform further functional analysis [36]. The seeds of the *Arabidopsis* Columbia ecotype (WT) and transgenic *Arabidopsis* were sterilized with 75% ethanol for 10 min and washed with deionized water three times. The sterilized seeds were placed on a medium containing 1/2 strength Murashige and Skoog (MS) containing 0.7% (*w*/*v*) agar and 2% (*w*/*v*) sucrose at a pH of 5.8–6.0. After two days of vernalization at 4 °C, they were placed in a light incubator for about 10 days. The seedlings were transplanted into pots filled with nutrient soil in the growing room and treated with drought and stress for three weeks under conditions of 22 °C, long photoperiods (16 h day/8 h night) and relative humidity of approximately 60%.

### 4.2. RNA Extraction and Quantitative Real-Time PCR

Total RNA was isolated using Trizol reagent (TIANGEN, Beijing, China) and the first cDNA strand was synthesized from 2 mg of total RNA using the FastKing RT Kit (Mona, China) [36]. The Light-CykerR96 Real-Time PCR system (BioRad, Hercules, CA, USA) was used for real-time quantitative PCR (qRT-PCR). All experimental data were obtained through three technical replicates, and the relative expression was calculated using the 2^−ΔΔCT^ method. The primer sequences used are listed in Table S1.

### 4.3. Germination Assay

To perform the characteristic analysis of seed germination under various abiotic stresses, the seeds of wild-type and transgenic *Arabidopsis* were firstly sterilized and uniformly germinated on a petri dish and grown in a controlled chamber (22 °C, 16 h light (120 μmol m^−2^s^−1^)/8 h darkness, and 60% relative humidity). For salt stress treatment, the petri dish was equipped with 1/2 MS solid medium containing 0 mM NaCl (serving as the control), 100 mM NaCl and 200 mM NaCl for seven days under salt stress, respectively. For drought stress treatment, the petri dish was equipped with 1/2 MS solid medium containing 0 mM mannitol (serving as the control), and 150 mM mannitol for seven days under drought stress, respectively. For ER stress treatment, the petri dish was equipped with 1/2 MS solid medium containing 0 mM DTT (serving as the control), 1 mM DTT, 2 mM DTT, and 5 mM DTT for 24 h under ER stress, respectively. Three biological replicates were conducted for the germination of Arabidopsis seeds in each treatment. After treatment, the germination potential and rate of seeds were counted in each treatment, respectively.

### 4.4. Salt, Drought and ER Stress Treatments

In order to treat salt stress, 3-week-old wild-type (WT) and transgenic *Arabidopsis* seedlings were placed in 200 mM and 400 mM NaCl, respectively, for 10 days. Then, the physiological characteristics and related gene expression of all the *Arabidopsis* lines were determined. For drought stress, seedlings of 3-week-old WT and transgenic *Arabidopsis* were subjected for 10 days to water deficiency, followed by one week of watering, and the plant phenotypes were recorded. For ER stress treatment, *Arabidopsis* seedlings with normal growth for 10 days were transplanted into 1/2MS plates containing 0 mM, 2 mM, and 5 mM DTT 24 h later. The phenotype and survival rate of *Arabidopsis* seedlings were observed on different concentrations of DTT plates. Moreover, four-week-old transgenic Arabidopsis and WT seedlings were evenly sprayed with 5 mM DTT and 10 mM DTT, respectively, and the phenotypes of the plants were recorded after 24 h of treatment.

### 4.5. Measurements of Physiological Indexes

The acetone extraction method was used to determine the contents of chlorophyll and carotenoid in leaves. The contents of soluble protein, proline, and MDA were also determined according to the kit instructions. In addition, the accumulations of H_2_O_2_ and O_2_^−^ in the leaves of the control and transgenic plants before and after heat stress were detected using 3,3′-diaminobenzidine (DAB) and Nitro blue tetrazolium (NBT), respectively. The H_2_O_2_ contents, activities of superoxide dismutase (SOD) and catalase (CAT) in stressed and control plants were determined using kits purchased from the Nanjing Jiancheng Bioengineering Institute (Nanjing, China).

### 4.6. Statistical Analysis

SPSS 20 software was used for data analysis; one-way ANOVA was used to analyze and compare the differences of the data of each group, and the new Duncan’s multiple range test difference method was used to compare the data of each group. Compared to the control group, *p* < 0.05 was considered to be statistically significant and *p* < 0.01 was considered extremely significant.

## 5. Conclusions

In conclusion, our results in this study demonstrate that *ZmNAC074* may be involved in the regulation of the abiotic stress response of *Arabidopsis* and some important physiological processes, thus enhancing the stress resistance of transgenic *Arabidopsis*. This study laid a foundation for further study of the function of *ZmNAC074* in maize, and provided an important basis for further functional analysis of the exact mechanism of crop stress resistance regulated by *ZmNAC074*. However, the special stress-resistant mechanism of *ZmNAC074* remains to be further elucidated.

## Data Availability

Data are contained within the article and Supplementary Materials.

## Supplementary Materials

The supporting information can be downloaded at: https://www.mdpi.com/article/10.3390/ijms242216157/s1.
