# Supplementary material for: Membrane-Bound Transcription Factor ZmNAC074 Positively Regulates Abiotic Stress Tolerance in Transgenic Arabidopsis
Source: Int J Mol Sci. 2023 Nov 10;24(22):16157. doi: 10.3390/ijms242216157 (PMC10671035; doi:10.3390/ijms242216157)
Supplement: Supplementary file 1 [file ijms-24-16157-s001.zip › ijms-2695357-supplementary.pdf]

Supplementary materials

Table S1 Primer sequences for RT-qPCR

Gene name	Forward primer sequence(5’ →3’)	Reverse primer sequence(5’ →3’)
ZmNAC074	GTGGACTACTACAAGTTCGACT	GTCGACGATCTTTCCAGACTTA
AtP5CS1	AGCTTGATGACGTTATCGATCT	AGATTCCATCAGCATGACCTAG
AtP5CS2	GTGCAAAACTGAATATTCCGGA	TTCTACAATTTCAACGGTGCAG
AtDREB2A	CATGTTTGATGTCGATGAGCTT	ATTCCGTAGTTGAGGCTTTGTA
AtDREB2B	GTATATGAACAAACCGGAACCG	ATACAACCTTTCTTCGACCCTT
AtAPX2	GAAATAGTTGCCTTGTCTGGTG	CATTTCACTGTCCATGACTGTC
AtbZIP60	GAAGGAGACGATGATGCTGTGGCT	AGCAGGGAACCCAACAGCAGACT
AtBZIP28	TTACTTGATCCAAGAGAGGTCG	AGGTAACGTACTTGACACTGTC
AtBIP3	CGTATAATCAATGAGCCAACGG	CCACCACCAAGATCATATACGA
AtPDI5	GATTCCAAGGATTTCGATGGTG	CAACATCGCCTTGGTATTAGTG
AtCNX1	CACCTGTGGAAAAGAAGAAACCAG	CAAGCAACCAGTTGTTACTACTG
AtActin1	CTCCTTTGTTGCTGTTGACTAC	GCACAATGTTACCGTACAGATC
